# Development of a novel adenovirus type 4 vector as a promising respiratory vaccine vehicle

**DOI:** 10.3389/fimmu.2025.1572081

**Published:** 2025-04-10

**Authors:** Jinghan Xu, Busen Wang, Zhenghao Zhao, Shipo Wu, Zhe Zhang, Shuling Liu, Nan Huo, Wanru Zheng, Yi Chen, Zhiqiang Gao, Zuyuan Jia, Tianyu Liu, Li Zhu, Lihua Hou

**Affiliations:** Laboratory of Advanced Biotechnology, Beijing Institute of Biotechnology, Beijing, China

**Keywords:** adenovirus, viral vector, vaccine, mucosal immunity, immune response

## Abstract

**Introduction:**

Adenovirus (Ad) vectors are widely used for gene delivery, and some of them have been approved for vaccine development. In particular, the recombinant COVID-19 vaccine for inhalation, which was developed using adenovirus type 5 (Ad5), represents a milestone in respiratory immunization. Owing to the high pre-existing immunity (PEI) to Ad5, the development of an adenoviral vector with lower PEI and higher immunogenicity has been explored. However, the majority of the developed novel Ad vectors showed suboptimal immunogenicity compared to Ad5 in animal models.

**Method:**

In this study, we constructed a novel replication-deficient viral vector based on human adenovirus type 4 (Ad4), which has long been used as a live virus vaccine with a favorable safety profile in the U.S. military. The mice were immunized intramuscularly or intranasally with an Ad4-vectored vaccine to verify immune responses and protective efficacy.

**Results:**

Compared with Ad5, the novel Ad4 vector showed comparable viral growth kinetics and transgene expression in cells and similar exogenous protein expression and distribution in mice. Furthermore, the Ad4-vectored vaccine elicited superior humoral and cellular responses and protective effects when vaccinated intranasally than those triggered by the Ad5-vectored vaccine. Finally, the heterologous Ad5 prime and Ad4 boost immunization showed better immunogenicity and protective efficacy.

**Discussion:**

This study broadens the research trajectory of adenovirus-vectored vaccines and offers a new option for the development of recombinant viral-vectored vaccines.

## 1 Introduction

In recent years, adenovirus (Ad) vectors have been widely used in the development of vaccines for emerging infectious diseases. Following the 2014 Ebola outbreak, adenovirus-vectored vaccines developed using the recombinant Ad5 (1, 2) and Ad26 (3) vectors were approved in China and Europe and showed good safety and immunogenicity. The chimpanzee adenovirus type 3-vectored Ebola vaccine (ChAd3-EBOZ) (4) has also entered phase II clinical trials involving thousands of participants. Ad-vectored vaccines have also played an important role in controlling the COVID-19 pandemic, with the recombinant Ad5-vectored COVID-19 vaccine (Ad5-nCoV) (5, 6) and the chimpanzee adenovirus-vectored ChAdOx1 nCoV-19 (7) being approved for use and Ad26.COV2.S (8) issued an Emergency Use Authorization.

Compared with inactivated vaccines, subunit vaccines, and mRNA vaccines, Ad-vectored vaccines can be delivered through the respiratory tract to generate mucosal antibodies and activate tissue-resident effector and memory T cells and resident memory B cells, which results in more effective respiratory pathogen prevention (9). These mucosal vaccines have been highlighted in recent clinical studies of COVID-19 vaccines. The aerosolized Ad5-vectored SARS-CoV-2 vaccine, which is delivered through mouth inhalation, induces a triple immune response encompassing cellular, humoral, and mucosal immunity; this vaccine exhibits high protective efficacy against SARS-CoV-2 infection and was approved for emergency use in China in September 2022 (10–12).

Although Ad5 exhibits high immunogenicity, pre-existing immunity (PEI) to Ad5 poses a challenge to its application. Previous studies have shown that high levels of anti-Ad5 neutralizing antibodies (NAbs) weaken the immune response, particularly the humoral immune response, and have a negative effect on the persistence of vaccine-elicited immune responses (2, 5). Furthermore, the widespread use of Ad-vectored vaccines, especially multiple booster doses of the same Ad-vectored vaccine during the pandemic, may lead to a higher immunopositivity rate or antibody levels against Ad5 or others, which could affect the immune response of follow-up vaccination (13, 14).

Several rare serotypes of adenovirus vectors have been developed for vaccines, but the majority of them do not have advantages in terms of immunogenicity over Ad5 vectors. For example, human Ads of rare serotypes, such as Ad6 (15), Ad11 (16), Ad24 (15), Ad26 (15–19), Ad28 (20, 21), Ad34 (15), Ad35 (15, 17, 19–21), Ad48 (16), Ad49 (16), and Ad50 (16) elicit lower antibody or cellular immune responses than Ad5. Furthermore, non-human adenovirus vectors, such as sAd11 (20, 21), sAd16 (20, 21), ChAd3 (20, 21), ChAd63 (20, 21), ChAdOx1 (22, 23), and AdC68 (22, 23), also elicit lower T cell or antibody responses than the Ad5 vector. Particularly at low doses of immunization, the immunogenicity and the protective efficacy of these rare serotypes of adenovirus vectors are significantly weaker than that of the Ad5 vector (15, 16, 18–20). Notably, a few rare serotypes of adenovirus have been used as respiratory vaccine vehicles, and there have been clinical results that revealed that intranasally delivered ChAdOx1 nCoV-19 failed to induce either a consistent mucosal antibody response or a strong systemic response (24). Under such circumstances, it is necessary to explore a novel Ad vector that can induce potent immune responses, not only systemic but also mucosal responses, similar to those induced by Ad5 with lower PEI.

In this study, we developed a novel rare serotype adenovirus type 4 vector with a high yield, and the protective efficacy and immunogenicity of a single intranasal dose of the Ad4-vectored vaccine were superior to those of an Ad5-vectored vaccine. Moreover, when used as a booster after Ad5 vectored-vaccine immunization, the Ad4-vectored vaccine induced more robust immune responses than the Ad5 homologous regimen. This study expands the platform of Ad-vectored vaccines and provides a new option for the development of recombinant vaccines.

## 2 Materials and methods

### 2.1 Cell culture

HEK293 cells (human embryonic kidney, ATCC), A549 cells (human non-small cell lung cancer cells, ATCC), and ACE2-293T cells (ACE2-expressing cell line, constructed by hygromycin B screening) were cultured at 37°C in a 5% CO_2_ incubator and maintained in Dulbecco’s modified Eagle’s medium (DMEM, Thermo Scientific, USA) supplemented with 10% fetal bovine serum (Thermo Scientific, USA), penicillin (100 units/ml) (Thermo Scientific, USA), and streptomycin (100 mg/ml) (Thermo Scientific, USA).

### 2.2 Animal ethical statement

Age-matched 6–8-week-old, specific pathogen-free (SPF) female BALB/c and hACE2 transgenic mice were purchased from Vital River Laboratories (Beijing, China) and Shanghai Model Organisms Center, Inc. (Shanghai, China), respectively. During animal experiments, such as intranasal immunization, the mice were anesthetized within a sealed chamber using isoflurane gas until they were completely unconscious. For tissue collection, the mice were humanely euthanized by CO_2_ asphyxiation followed by cervical dislocation. The animal experiments were conducted in compliance with the protocols established by the Institutional Experimental Animal Welfare and Ethics Committee.

### 2.3 Construction of the recombinant Ad4 vector

The replication-deficient Ad4 vector was constructed based on the E3 region-deleted replication vector in our previous study (25). The plasmid was subjected to double-enzyme digestion with *Pme*I and *Nde*I to delete the E1 region. Afterward, we added 450 bp (hereafter referred to as Ad4-RI67) to produce a new *Pme*I site, and the original *Pme*I site was mutated. A Gibson assembly kit (New England Biolabs, USA) was used to construct an E1/E3-deleted replication-deficient Ad4 vector plasmid. The same method was used to modify the E4 region, except that the *Spe*I and *Asis*I restriction enzymes were used, and to insert exogenous genes, the spike protein of the WT Wuhan-Hu-1 strain (NC_045512.2) or the luciferase reporter gene and the expression cassette between the packaging signal sequence and the original E1 region were generated using the newly produced *Pme*I site. The recombinant plasmids were linearized via the *Pac*I enzyme and transfected into HEK293 cells using TurboFect transfection reagent (Thermo Fisher Scientific, USA). The transfected cells were passaged when they were overgrown and collected until cytopathic effects were observed. The cells were lysed through three freeze-thaw cycles to release the recombinant viruses.

The infectious units were titrated on HEK293 cells using the same method with an Adeno-X™ Rapid Titer Kit (Clontech, USA), with the exception that the primary and secondary antibodies were mouse anti-adenovirus Hexon (AbD Serotec, Kidlington, UK) and goat anti-mouse IgG2a (Abcam, Cambridge, UK) (25).

### 2.4 Replication growth curve of the recombinant adenovirus

A549 cells were cultured in 12-well cell plates (2.5×10^5^ cells per well) for 16 h and respectively infected with recombinant adenovirus with a multiplicity of infection (MOI) of 1. After infection for 2 h, the cell culture medium was replaced and washed twice with phosphate-buffered solution (PBS). At 6,12, 24, 48, 72, and 96 h post-infection, the cell suspension was collected and subjected to three cycles of freeze-thaw at 37°C and -80°C. After centrifuging, the supernatant was collected. Viral titers from different time points were determined using the standard method with HEK293 cells for non-replicating adenovirus (25). The viral replication growth curves were plotted using the log10-transformed viral titers at different time points post-infection. The viral replication growth curve in the HEK293 cells was detected using the same method, but 5×10^5^ cells were seeded per well so that the cell confluence reached the same level before viral infection. The viral replication growth curves were constructed using the GraphPad Prism software.

### 2.5 Western blotting

HEK293 and A549 cells were seeded in six-well cell culture plates and transiently transfected with recombinant adenovirus-vectored vaccines at an MOI of 1. At 24 h post-transfection, the culture supernatant was discarded, and the cells were lysed with 200 μl of RIPA lysis buffer. Then, the cell lysate supernatant was mixed with a protein loading buffer (Thermo Scientific, USA). Western blotting was performed on the samples with a SARS-CoV-2 spike antibody (Sino Biological, China), followed by goat anti-rabbit IgG (with HRP, Cell Signaling Technology). An anti-β-actin antibody was used as an internal control (with HRP, Abcam, Cambridge, UK).

### 2.6 Animal immunization and SARS-CoV-2 challenge

The BALB/c mice were randomly divided and immunized intramuscularly (thigh muscle of the hind limb) or intranasally (under anesthesia). For single-dose immunization, the mice were immunized with 5×10^5^, 1×10^6,^ and 1×10^7^ infection units (IFUs) via the intramuscular (i.m.) and intranasal (i.n.) routes, respectively. Mice were immunized with 1×10^6^ IFUs via the i.m. or i.n. route on day 0 (prime) and day 28 (boost) for the prime-boost immunization. Blood was collected at various time points post-immunization, and tissues were harvested from humanely euthanized mice. Serum, bronchoalveolar lavage fluid (BALF), and nasal lavage fluid (NLF) samples were collected to measure spike-specific binding antibodies and pseudovirus-neutralizing antibodies. Spleen and lung samples were collected for analysis of cellular immune responses.

The hACE2 transgenic mice were also randomly divided and immunized intramuscularly or intranasally with 1×10^6^ or 1×10^7^ IFUs for single-dose immunization or with 1×10^6^ IFUs for prime-boost immunization (interval of 28 days). At 28 days post-immunization and 14 days post-booster, the hACE2 transgenic mice were intranasally administered 1×10^4^ TCID_50_ SARS-CoV-2. On the third day after the challenge, the mice were euthanized to collect lung and turbinate tissues to assess the tissues’ viral load. The SARS-CoV-2 challenge experiment was conducted by the National Vaccine and Serum Institute of China in a biosafety level 3 laboratory.

### 2.7 ELISA

Spike-specific IgG and IgA antibody levels in mouse serum, BALF, and NLF were measured by ELISA. ELISA plates were coated with 1 μg/ml antigen proteins at 4°C overnight with S proteins obtained from Sino Biological (Beijing, China). After blocking, serial dilutions of serum were added to the plates, which were subsequently incubated at 37°C for 1 h. HRP-conjugated goat anti-mouse IgG or IgA (Abcam, UK, 1:10,000 dilution) was added to the plates. The plates were again incubated at 37°C for 1 h, after which TMB substrate solution (Solarbio, China) was added, and the reaction was stopped by the addition of stop solution (Solarbio, China). Finally, the optical density (OD) at 450–630 nm was recorded with a microplate reader (SPECTRA MAX 190, Molecular Devices, USA). The endpoint titer was defined as the highest reciprocal serum dilution that yielded an absorbance ≥2.1-fold over the negative control serum values. The IgA-binding antibody titer below the limit of detection of 30 was assigned a value of 15. The IgG-binding antibody titer of BALF below the limit of detection of 50 was assigned a value of 25, while the IgG binding antibody titer of NLF below the limit of detection of 10 was assigned a value of 5.

### 2.8 Pseudovirus neutralizing assay

SARS-CoV-2 pseudoviruses were purchased from Vazyme (Nanjing, China). To determine the neutralizing activity of mouse serum, heat-inactivated serum was serially diluted threefold at a 1:30 dilution ratio and mixed with an equal volume of pseudovirus in 96-well plates. After 1 h of incubation at 37°C, ACE2-293T cells were added. After 48 h, the activity of luciferase was measured via a Bright-Life Luciferase Assay System (Vazyme, Nanjing, China). The EC_50_ pseudovirus-neutralizing antibodies (pNAbs) were calculated as the reciprocal of the dilution at which luciferase activity was reduced by half with the virus control. For calculations of geometric mean titer (GMT), titers below the detection limit of the assay were assigned a value of half of the initial dilution.

### 2.9 *In vivo* luciferase activity

To detect Ad4 infection *in vivo*, BALB/c mice (n = 5) were intramuscularly or intranasally administered Ad4-luc-E or Ad5-S at a dose of 10^7^ IFUs. At 6 h and 24 h post-inoculation, the animals were intraperitoneally injected with a luciferase substrate (Perkin Elmer, MA, USA). After reacting for 10 min, fluorescence signals were detected via an IVIS Spectrum instrument, and the fluorescence signals were quantified using Living Image 3.0.

### 2.10 T-cell response by ELISpot

The specific IFNγ-secreting T-cell response was measured via an enzyme-linked immunospot (ELISpot). Mouse spleens were ground and filtered through a 70 μm cell strainer to create a single-cell suspension. The lungs were cut into small pieces and digested with collagenase type IV (2 mg/mL, Roche, Basel, Switzerland) and DNase I (0.1 mg/mL, Solarbio, Beijing, China) for 45 min at 37°C, followed by filtering through 70-pum strainers to make a single-cell suspension. Mouse splenocytes (2×10^5^ cells/well) and lung cells (1×10^5^ cells/well) were stimulated with S peptides. The SARS-CoV-2 S antigen-specific T-cell response in mice was assessed by IFNγ ELISpot Kits (Mabtech, Nacka Strand, Sweden) following the manufacturer’s instructions. The spots were counted with an AT-Spot 3200 (SinSage Technology, Beijing, China). The results are expressed as the number of SARS-CoV-2-specific spots per million cells.

### 2.11 Intracellular cytokine staining assay

Splenocytes or homogenized lung cells were stimulated for 8 h with S-specific overlapping peptide pools (1 µg/ml of each peptide) in the presence of Golgistop™, which inhibits cytokine secretion (4 μg/mL, BD, USA). Then, the cells were washed and stained with anti-CD16/32 antibodies and NIR viability dye (Thermo Fisher Science, USA) on ice for 20 min to rule out non-specific binding and dead cells. After being washed, the cells were incubated with a mixture of antibodies against lineage markers, such as anti-CD3 PerCP-Cy5.5, anti-CD4 Alexa Fluor 700, anti-CD8 BV510, anti-CD14 APC/Cy7, and anti-CD19 APC/Cy7. After being washed with PBS, the cells were fixed and permeabilized with Cytofix/Cytoperm, washed with 1× PermWash buffer, and then incubated with anti-IFNγ PE, anti-IL2 BV421, and anti-TNFα PE-Cy™7 antibodies. The cells were washed with Perm/Wash buffer and PBS. All mAbs and reagents were purchased from BD Biosciences (San Diego, CA, USA). Data were acquired on a FACS CantoTM and analyzed using FlowJo v10.

### 2.12 SARS-CoV-2 viral load determination

In total, 100 mg of mouse lung or turbinate tissue was weighed and homogenized. Viral RNA was isolated, and reverse transcription-quantitative PCR (RT–qPCR) assays to detect the N gene of the viral genome were performed using the SuperScript^®^ III One-Step RT-PCR Kit (Thermo Fisher Scientific, USA). Viral loads were expressed on a log10 scale as viral copies/mg after being calculated with a standard curve. Data below the limit were set at a value of 5 copies/mg.

### 2.13 Statistical analysis

The analysis was performed using GraphPad Prism v.8.00. Unpaired t-tests were conducted to compare differences between the two experimental groups. One-way ANOVA with Tukey’s multiple comparisons test was used to compare more than two experimental groups. *p < 0.05, **p < 0.01, and ***p < 0.001 were considered to indicate significance. The antibody titer data were log-transformed before analysis. The error bars in all figures represent one standard deviation.

## 3 Results

### 3.1 Construction of the E1/E3-deleted and E4-modified Ad4 vectors

**Figure 1:**
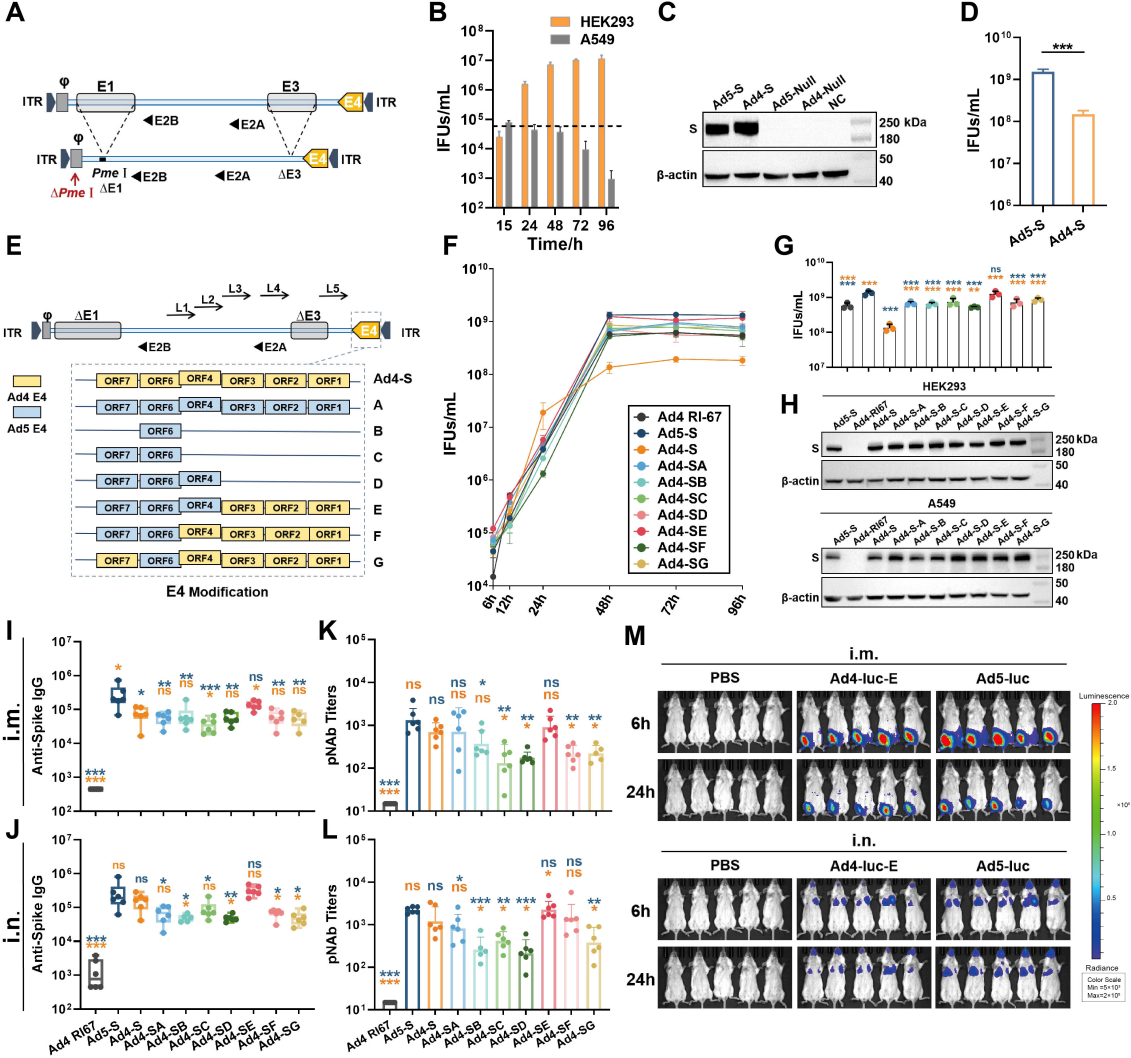
Vector design and immunogenicity induced by the modified Ad4 vectors. **(A)** Schematic of the construction of the E1/E3-deleted Ad4 vector. **(B)** The replication capacity of the replication-deficient Ad4 vector in HEK293 and A549 cells. The dashed line represents the infection titer. **(C)** The transgene expression levels of Ad4-S and Ad5-S SARS-CoV-2 vaccine candidates in HEK293 cells. The expression of SARS-CoV-2 Spike was validated through western blotting. Ad5-null and Ad4-null were empty vectors with no antigen genes. **(D)** The viral infection units (IFUs) of Ad4-S and Ad5-S in HEK293 cells. **(E)** Schematic of the seven strategies of recombinant E4-modified Ad4 vectors. The vectors replaced the whole or partial E4 regions of Ad5 and referred to as Ad4-SA to SG. **(F)** The growth curve of these recombinant adenoviruses. Ad4 RI67, Ad5-S, Ad4-S, and Ad4-SA to SG were infected in HEK293 cells, and IFUs were detected at 6, 12, 24, 48, 72, and 96 h after infection. **(G)** The IFUs of the vectors in HEK293 cells at 48h after infection. **(H)** The transgene expression levels of the modified Ad4 vectors. The expression level of SARS-CoV-2 spike protein was detected by Western blotting in HEK293 and A549 cells 24h post-infection at an MOI of 1. **(I–L)** The immunogenicity of the modified Ad4 vectors. BALB/c mice (n=6 per group) were i.m. or i.n. immunized with 1×10^7^ IFUs, IgG binding antibody titers **(I, J)** were detected by ELISA, and pseudovirus neutralizing antibody (pNAb) titers **(K, L)** were detected by using HIV backbone-derived pseudovirus at day 28 post-immunization. **(M)** *In vivo* expression and distribution of the exogenous protein encoding. BALB/c mice (n=5 per group) were i.m. or i.n. immunized with 1×10^7^ IFUs of Ad4-luc-E or Ad5-luc, or with PBS as a control. Luciferase activity was imaged at 6 h and 24h after immunization on the IVIS Spectrum imaging system. Signals ranged from low activity (shown in blue) to high activity (shown in red) levels. The bar graphs show the GMT values **(I–L)** with a 95% confidence interval (CI) or mean **(B, D, F, G)** with SEM values. Statistical significance was determined by one-way ANOVA with Tukey’s multiple comparisons tests among E4-modified groups (only showing a significant difference compared to Ad5-S or Ad4-S) and by a two-tailed t-test between Ad4-S and Ad5-S. *** p < 0.001, ** 0.001<p < 0.01, * 0.01<p < 0.05 and ns for p > 0.05.

In a previous study, we constructed an E3-deleted replication-competent human Ad4 vector (Ad4-dE3) based on the ATCC Ad4-RI67 strain (GenBank No. AY594253) (25, 26). Here, we deleted the E1 region of pAd4-dE3 (plasmid of Ad4-dE3 vector) to construct a replication-deficient Ad4 vector (Ad4-dE1/E3) for improved safety. Additionally, we mutated the nucleotide base to delete the original *Pme*I site in the packaging signal region and added an extra *Pme*I site behind the packaging signal in front of the E1 region to insert the exogenous gene (**Figure 1A**). As a model antigen, the SARS-CoV-2 spike gene expression cassette was inserted into the *Pme*I site of Ad4-dE1/E3 to construct an Ad4-vectored vaccine (Ad4-S). When cells were infected with Ad4-S at an MOI of 1, the titer of Ad4-S in A549 cells decreased over time but increased gradually and almost peaked at 48 h in HEK293 cells (**Figure 1B**), which demonstrated that Ad4-dE1/E3 lost the ability to replicate in common cells but retained replication capability in E1-containing HEK293 cells. HEK293 cells were infected with Ad4-S or Ad5-S at an MOI of 1, the expression of the SARS-CoV-2 spike protein was validated through Western blotting at 24h after infection, and the IFUs were measured by a Hexon Immunoassay at 48h after infection. Ad4-S effectively expressed the spike protein in HEK293 cells, and the expression level was similar to that of Ad5-S (**Figure 1C**), but the viral titers of Ad4-S were approximately 10-fold lower than those of Ad5-S (**Figure 1D**).

Modification of the E4 region is necessary for the efficient propagation of some non-Ad5 vectors in HEK293 cells (27). To increase the productive capacity of the Ad4 vector, we replaced different E4 regions of Ad4-S with those of Ad5 to construct seven types of E4-modified recombinant viruses, referred to as Ad4-SA to -SG (**Figure 1E**). Ad4-S (E4 unmodified), Ad5-S as a positive control, and Ad4-RI67 (adenovirus type 4 wild-type) as a negative control were used. Infection of modified Ad4-S in HEK293 cells at an MOI of 1 significantly increased the growth curve of those E4-modified Ad4 vectors, similar to that of the Ad5 vector (**Figure 1F**). The viral titers at 48 h after infection were 4–9-fold greater than those of the E4-unmodified vector (**Figure 1G**). Compared with Ad5-S, the spike protein expressions of Ad4-SA to -SG were comparable in HEK293 and higher in A549 cells (**Figure 1H**; **Supplementary Figure S1**). Among them, the replacement of ORF4,6/7 with Ad5 (Ad4-SE) almost achieved the highest viral titer and exogenous protein expression capacity.

### 3.2 Immunogenicity after immunization with the novel Ad4-vectored vaccines and exogenous protein distribution *in vivo*

E4 is associated with apoptosis (28), which may affect antigen gene expression and the antibody response. To understand the antibody response of E4-modified Ad4 variant vectors with spike as a model antigen, BALB/c mice were immunized intramuscularly or intranasally with 10^7^ IFUs of Ad4-SA to -SG or with the same dose of Ad5-S, Ad4-S, or Ad4-RI67 as controls. All groups were induced with spike-specific serum IgG antibodies and pNAbs on day 28 after immunization (**Figures 1I–L**). Among the E4-modified Ad4 vectors, Ad4-SE induced the highest level of serum IgG and pNAbs, which was comparable to that of Ad5-S. The expression and distribution of exogenous protein *in vivo* were also assessed based on the E4 ORF4,6/7-replaced Ad4 recombinant virus encoding the luciferase gene (Ad4-luc-E). BALB/c mice were inoculated i.m. or i.n. with 10^7^ IFUs of Ad4-luc-E or Ad5-luc or with PBS as a control, and luciferase activity was imaged at 6 h and 24 h. Ad4-luc-E, whether administered i.m. or i.n., induced comparable luciferase expression levels and distributions to those of Ad5-luc (**Figure 1M**).

### 3.3 A single dose vaccination of Ad4-SE via the intranasal route induces superior antibody and T-cell responses to those of Ad5-S in mice

**Figure 2:**
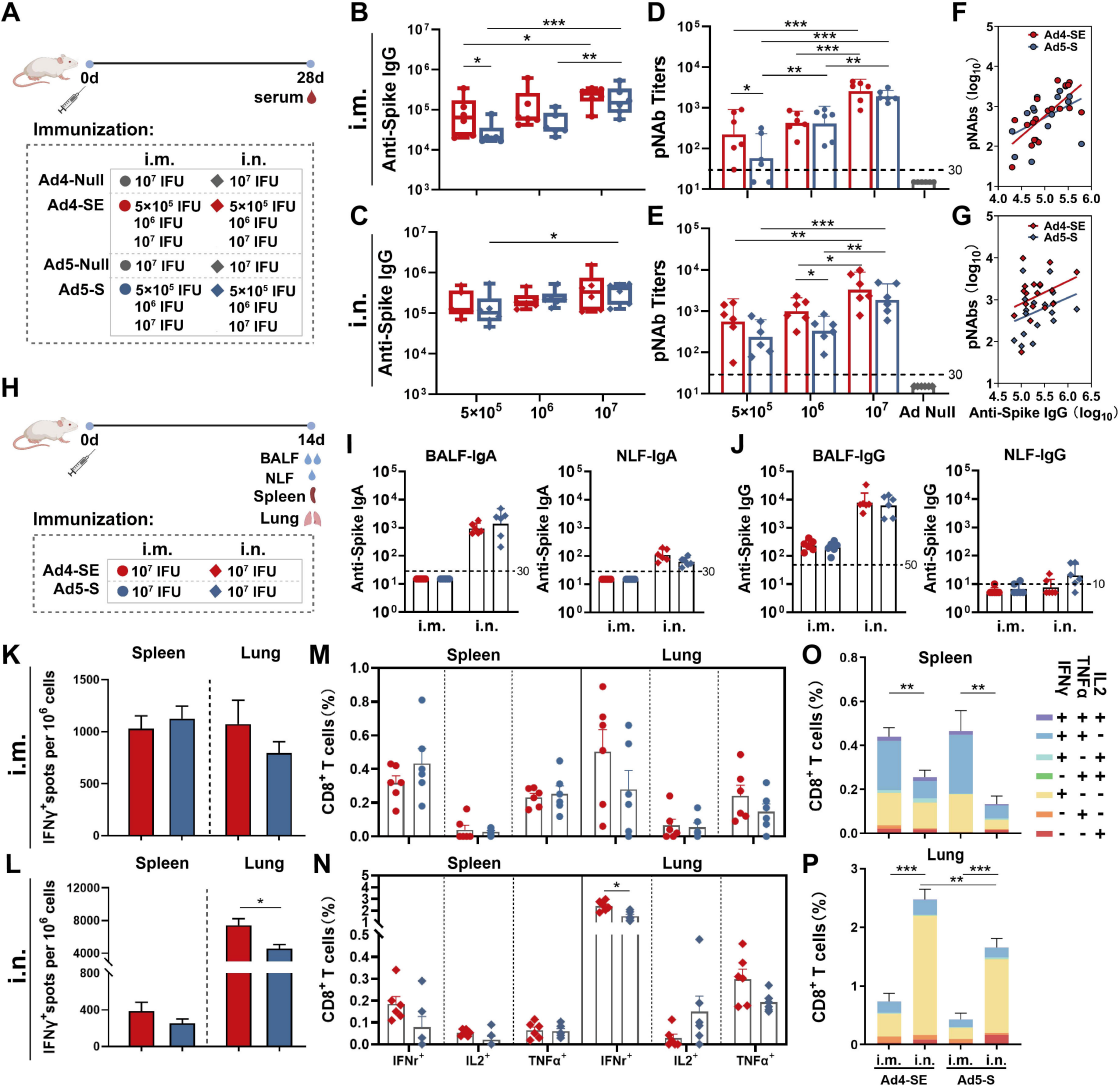
Humoral and cellular immune responses of Ad4-SE and Ad5-S. **(A)** Schedule of immunization and sampling for serum antibody detection. BALB/c mice (n = 6 per group) received a single dose via i.m. or i.n. immunization of 5×10^5^, 1×10^6^, or 1×10^7^ IFUs of Ad4-SE or Ad5-S, or 1×10^7^ IFUs of Ad4-Null or Ad5-Null as controls. Serum was collected on day 28. **(B–E)** Anti-spike IgG binding antibodies and the pNAbs of the i.m. **(B, D)** and i.n. **(C, E)** were detected. **(F, G)** The correlation of anti-spike IgG binding antibodies and pNAbs in the i.m. **(F)** and i.n. **(G)** groups. **(H)** Schedule of immunization and sampling for mucosal antibody detection and intracellular cytokine assay. BALB/c mice (n = 6 per group) received a single dose i.m. or i.n. immunization with 1×10^7^ IFUs of Ad4-SE or Ad5-S. The BALF, NLF, spleen, and lungs were harvested on day 14 post-immunization. **(I,J)** The anti-spike IgA titers **(I)** and IgG titers **(J)** of the BALF and NLF were detected by ELISA. **(K–N)** IFNγ-producing T-cell responses in splenocyte and lung cells were detected by ELISpot in the i.m. **(K)** and i.n. **(L)** groups. The reactive T cells were presented as spot-forming cells (SFCs)/million cells. IFNγ-, IL2-, and TNFα-secreting CD8^+^ T-cells in the spleen and lungs of the i.m. (**M**) and i.n. **(N)** groups were measured by flow cytometry. **(O, P)** The proportions of CD8^+^ T-cells that produce one, two, and three cytokines in the spleen **(O)** and lungs **(P)**. The dotted lines in the figure represent the detection limit. The bar graphs show the GMT values **(B–E, I, J)** with 95% confidence interval (CI) or mean **(K–P)** with SEM values, statistical significance was determined by one-way ANOVA with Tukey’s multiple comparisons tests among the dose escalation groups and by a two-tailed t-test between Ad4-SE and Ad5-S. *** p < 0.001, ** 0.001<p < 0.01, * 0.01<p < 0.05.

To evaluate the humoral immune response of the novel Ad4-vectored vaccine, BALB/c mice were immunized with 5×10^5^, 1×10^6^, or 1×10^7^ IFUs of Ad4-SE or Ad5-S via the i.m. or i.n. route with 1×10^7^ IFUs of Ad4-Null or Ad5-Null as controls, and serum samples were collected on day 28 after immunization for antibody detection (**Figure 2A**). Both Ad4-SE and Ad5-S induced dose-dependent anti-spike IgG and SARS-CoV-2 pNAb responses (**Figures 2B–E**). In addition, Ad4-SE induced slightly higher antibody titers than Ad5-S in almost all comparison groups, and significant differences in pNAbs exist in the i.m. groups treated with the 5×10^5^ IFU dose and the i.n. groups treated with the 1×10^6^ IFU dose. An obvious correlation was evident between pNAbs and anti-spike IgG antibodies (**Figures 2F–G**). At the same level of anti-spike IgG titers, the Ad4-SE groups showed higher pNAbs than the Ad5-S groups when immunized via the i.n. route (**Figure 2G**).

To evaluate the mucosal antibody and cellular immune response of Ad4-SE, BALB/c mice were immunized with 1×10^7^ IFUs of the vaccine via the i.m. or i.n. route, and samples were collected 14 days after immunization (**Figure 2H**). The same dose of Ad5-S was used as a control. Anti-spike IgA antibodies in the BALF and NLF were observed in the i.n. mice but not in the i.m. mice (**Figure 2I**). Anti-spike IgG was observed in the BALF of all the groups, with significantly higher levels in the i.n. groups than in the i.m. groups (**Figure 2J**).

Splenocytes and lung cells were collected, and cellular immune responses were validated using IFNγ ELISpot and intracellular cytokine staining for IFNγ, IL2, and TNFα (**Supplementary Figure S2**). The result of intracellular cytokine staining correlated well with the IFNγ ELISpot assay. Ad4-SE elicited a robust CD8 biased cellular immune response when immunized i.m., characterized by a predominant IFNγ^+^ and IFNγ^+^TNFα^+^ response in the spleen and a predominant IFNγ^+^ response in the lungs, showing no significant difference compared with Ad5-S (**Figures 2K, M, O, P**; **Supplementary Figure S3**). When administered intranasally, both the Ad4-SE and Ad5-S groups exhibited a lower level of IFNγ^+^ response in the spleen, and a higher level of IFNγ^+^ response in the lungs was observed than those observed with the i.m. vaccination regimen. Notably, the Ad4-SE i.n. immunization elicited a significantly higher cellular immune response in the lungs, especially the CD8^+^ IFNγ^+^ response, compared to Ad5-S (**Figures 2L, N**).

### 3.4 Intranasal immunization with the Ad4-vectored vaccine effectively protects mice from viral infection in both the upper and lower respiratory tracts

**Figure 3:**
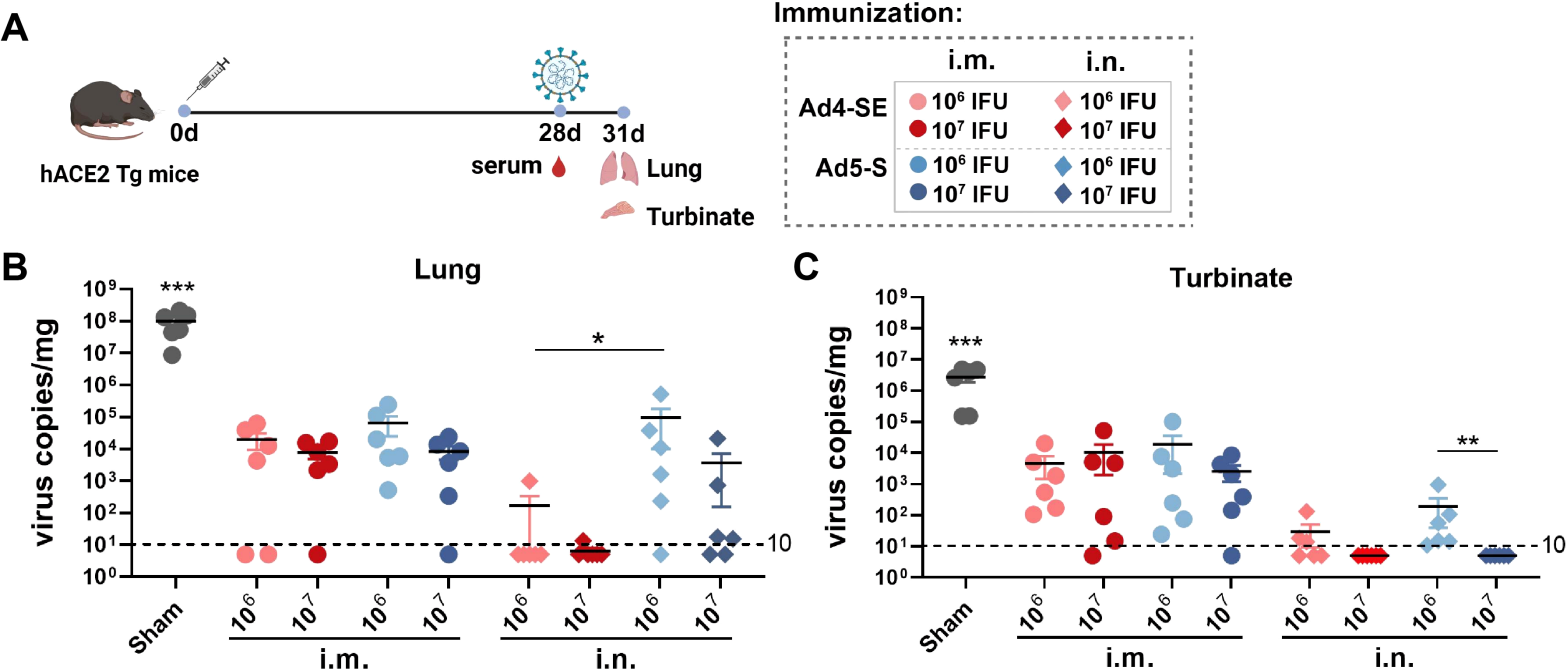
Single-dose immunization protection against SARS-CoV-2 in hACE2 transgenic mice. **(A)** Schedule of immunization, challenge, and sampling. The hACE2 mice (n = 6 per group) received a single dose with 1×10^6^ or 1×10^7^ IFUs of Ad4-SE or Ad5-S via i.m. or i.n. immunization. Mice that received the same volume of PBS were set as the sham group. Mice were challenged with 1×10^4^ TCID_50_ of SARS-CoV-2 on day 28 and sacrificed to collect lung and turbinate tissues 3 days post-challenge. Serum was also collected on day 28 before the challenge. **(B, C)** Viral gRNA copies in the lungs **(B)** and the turbinates **(C)** were detected by RT-qPCR. The dotted lines in the figure represent the detection limit. Data are shown as mean ± SEM. Statistical significance among the different groups was determined by a two-tailed t-test. *** p < 0.001, ** 0.001<p < 0.01, * 0.01<p < 0.05.

To evaluate the protective effects of Ad4-SE, we immunized hACE2 transgenic mice with a single dose of 1×10^6^ or 1×10^7^ IFUs via the i.m. or i.n. route; the PBS group was used as a control. The vaccinated mice were challenged on day 28 with 1×10^4^ TCID_50_ SARS-CoV-2 and sacrificed to collect lung and turbinate tissues for viral gRNA detection on day 3 post-challenge (**Figure 3A**). Compared with those in the negative control group (sham), the viral gRNA loads in all vaccine groups were significantly lower (**Figures 3B, C**). Intranasal immunization clearly provided better respiratory protection than intramuscular immunization as the IgG antibody responses and pNAbs of the i.n. groups were not better than those of the i.m. group (**Figure 3**; **Supplementary Figure S4**). The viral load at 1×10^7^ IFUs was lower than that at 1×10^6^ IFUs via the i.n. route, and all the i.n. groups in the Ad4-vector group provided better protection than the Ad5-vector group did, especially in the lungs (**Figures 3B, C**). Correspondingly, the IgG antibody response in the serum of the i.n. group was significantly stronger in the high-dose group than in the low-dose group. The Ad4 vector induced a stronger antibody response than Ad5 did, although there were no significant differences, except for the effect of the 1×10^6^ dose of anti-spike IgG (**Supplementary Figure S4B**). These results suggested that the novel Ad4-vectored vaccine could effectively induce mucosal immune responses and had a better protective effect than the Ad5-vectored vaccine.

### 3.5 Heterologous immunization with Ad4-SE has significant advantages in inducing immune responses

**Figure 4:**
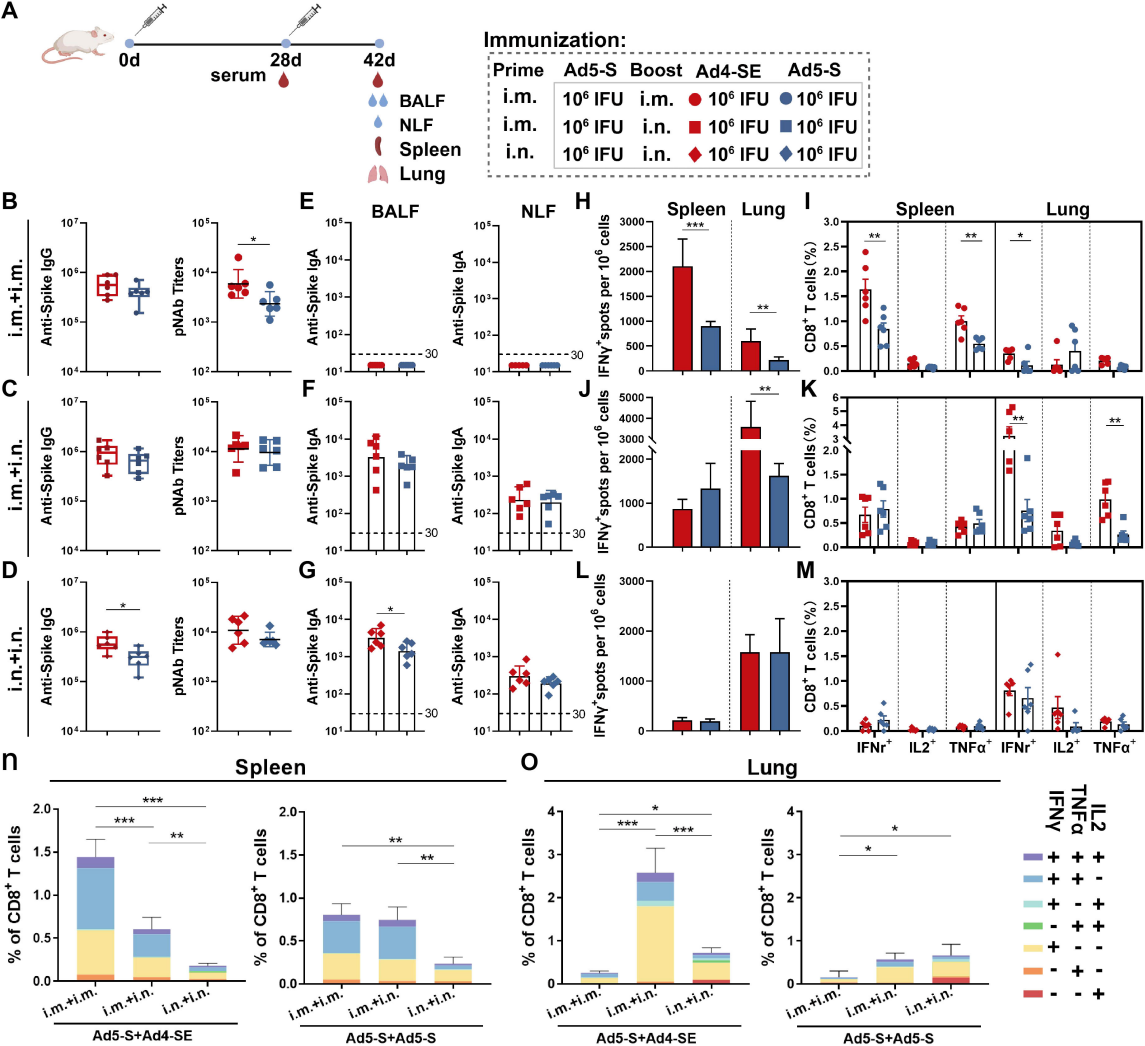
Humoral and cellular immune responses induced by Ad4-SE heterologous or Ad5-S homologous booster vaccination. **(A)** Schedule of immunization and sampling. BALB/c mice (n = 6 per group) were primed with 1×10^6^ IFUs of Ad5-S through the i.m. or i.n. route and boosted with the same dose of Ad4-SE or Ad5-S via i.m. or i.n. immunization on day 28. Serum was collected on day 28 and day 42. The BALF, NLF, spleen, and lungs were harvested on day 42. **(B–D)** Serum IgG-binding antibodies and pNAbs on day 42 in the i.m.Prime-i.m.Boost (i.m.+ i.m.) group **(B)**, i.m.Prime-i.n.Boost (i.m. + i.n.) group **(C)**, and i.n.Prime-i.n.Boost (i.n. + i.n.) group **(D)**. **(E–G)** IgA binding antibodies in the BALF (left) and NLF (right) on day 42 in the i.m. + i.m. group **(E)**, i.m. + i.n. group **(F)**, and i.n. + i.n. group **(G)**. **(H–M)** Spike-specific IFNγ spots in the spleen and lungs on day 42 in the i.m. + i.m. group **(H)**, i.m. + i.n. group **(J)**, and i.n. + i.n. group **(L)**. The reactive T cells were presented as spot-forming cells (SFCs)/million cells. Spike-specific IFNγ, IL2, and TNFα secreting CD8^+^ T-cell response in the spleen and lungs on day 42 in the i.m. + i.m. group **(I)**, i.m. + i.n. group **(K)**, and i.n. + i.n. groups **(M)**. **(N, O)** The proportions of CD8^+^ T-cells that produce one, two, and three cytokines in the spleen **(N)** and lungs **(O)**. The dotted lines in the figure represent the detection limit. The bar graphs show the GMT values with 95% CI **(B–G)** and mean values with SEM **(H–O)**. Statistical significance was determined between Ad4-SE and Ad5-S by a two-tailed t-test and by one-way ANOVA with Tukey’s multiple comparisons tests among different prime-boost strategy groups. *** p < 0.001, ** 0.001<p < 0.01, * 0.01<p < 0.05.

We further analyzed the immune response of different prime-boost regimens, such as the heterologous Ad5-S-Prime Ad4-SE-Boost and the homologous Ad5-S-Prime Ad5-S-Boost regimens. Mice were initially immunized with 1×10^6^ IFUs of Ad5-S intramuscularly or intranasally and received the same doses of the Ad4-SE vaccine or Ad5-S vaccine administered intramuscularly or intranasally on day 28 after priming. Serum, BALF, and NLF were collected to evaluate antibody responses on day 42 (**Figure 4A**).

All immunization regimens elicited significantly robust antibody responses, and the heterologous regimen with the Ad4-SE booster induced higher antibody titers (**Figures 4B–D**). In addition, the effect of an extended time interval between priming and boosting on the immune response was explored, and it was observed that after 18 months, the increase in antibody levels with heterologous boosting was higher than that with homologous boosting, significantly in the i.m+i.n. and i.n.+i.n. groups (**Supplementary Figure S5**). The detection of spike-specific IgA responses in the BALF and NLF revealed that i.m. immunization did not induce mucosal IgA responses (**Figure 4E**). In the BALF, the IgA responses induced by heterologous immunization with the Ad4-SE in intranasal boost groups were higher than those induced by Ad5-S homologous immunization (**Figures 4F, G**).

In the i.m.Prime-i.m.booster regimen, spike-specific IFNγ-producing ELISpot, CD8^+^ IFNγ^+^, and CD8^+^ TNFα^+^ T cell responses were significantly higher in the spleen than in the lungs (**Figures 4H, I**). However, when the Ad-vectored vaccine was boosted via i.n. route, higher cellular responses in the lungs were observed than in the spleen (**Figures 4J–M**), and the lowest T cell response in the spleen was observed in the two-dose intranasal immunization regimen (**Figures 4L, M**). Additionally, heterologous boosting with the Ad4-SE vaccine demonstrated superior T cell response compared with homologous boosting using the Ad5-S, especially in i.m.Prime-i.m.Boost or in i.m.Prime-i.n.Boost regimens. Among all groups, the i.m.Prime-i.m.Boost heterologous regimen induced the highest IFNγ^+^ and TNFα^+^ cellular response in the spleen, while the i.m.Prime-i.n.Boost heterologous regimen elicited the highest T cell response in the lungs (**Figures 4N, O**). Similar results were observed for CD4^+^ T-cell responses in the lungs (**Supplementary Figure S4**). Polyfunctional analysis also revealed that the main proportion of cytokine-positive CD8^+^ T cells was consistent with single-dose immunization (**Figures 4N, O**; **2O, P**).

### 3.6 Ad4-vectored vaccine heterologous boosting via the i.m.Prime-i.n.Boost regimen showed the best protective effects

**Figure 5:**
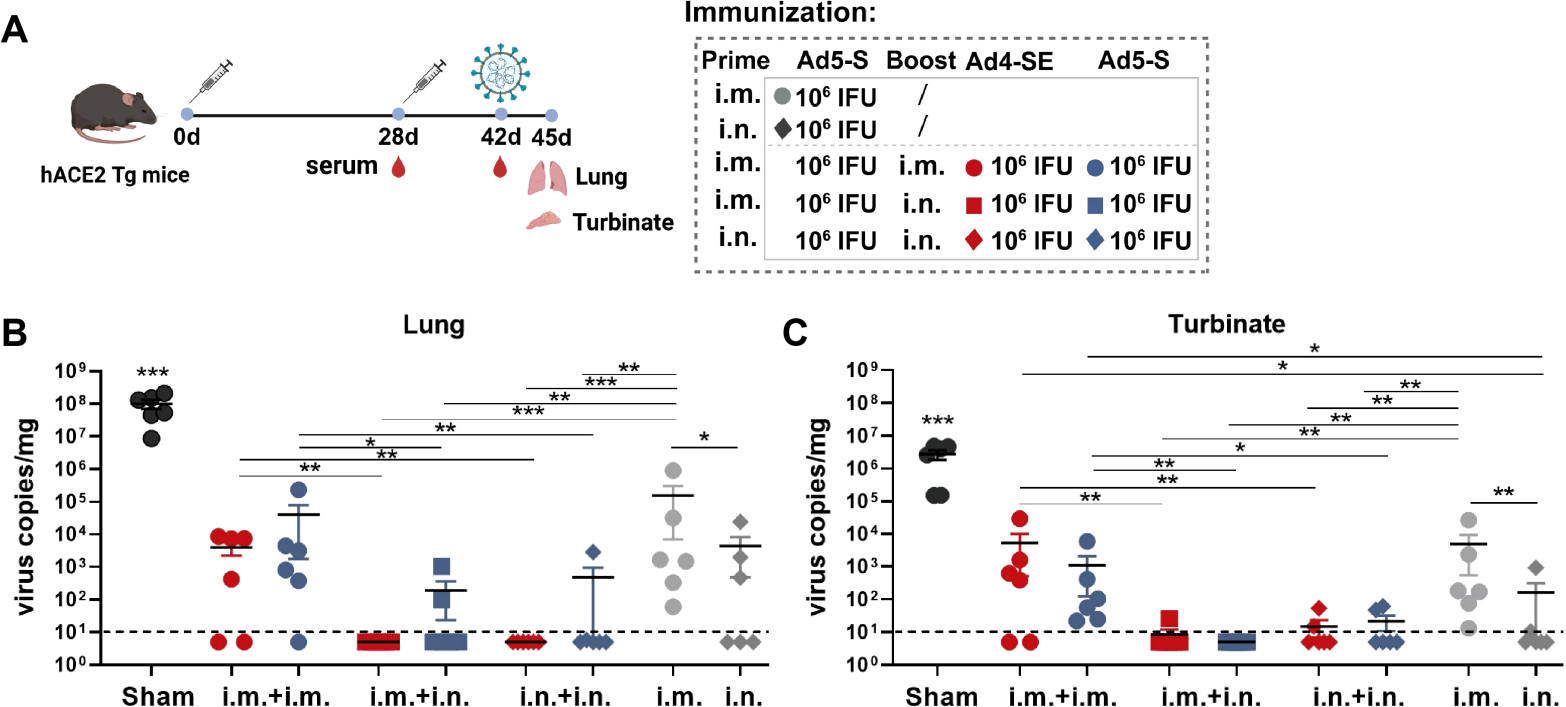
Ad4-SE heterologous booster vaccination provided great protection against SARS-CoV-2 in hACE2 transgenic mice. **(A)** Schedule of immunization, challenge, and sampling. hACE2 mice (n = 6 per group) were primed with 1×10^6^ IFUs of Ad5-S through the i.m. or i.n. route and boosted with the same dose of Ad4-SE or Ad5-S via i.m. or i.n. immunization on day 28, or without booster as controls. Mice were challenged with 1×10^4^ TCID_50_ of SARS-CoV-2 on day 42 and sacrificed to collect lung and turbinate tissues after 3 days post-challenge. Serum was also collected on day 28 (before boosting) and day 42 (before challenge). **(B, C)** Viral gRNA copies in lungs **(B)** and turbinates **(C)** were determined through RT-qPCR. The dotted lines in the figure represent the detection limit. Data are shown as mean ± SEM, and statistical significance was determined by one-way ANOVA with Tukey’s multiple comparisons tests among the different prime-boost regimens and by a two-tailed t-test between Ad4-SE and Ad5-S.

We next evaluated the protective effects of Ad4-SE in a prime-boost regimen by intramuscular or intranasal immunization in hACE2 mice. The single-dose group was immunized intramuscularly or intranasally with the Ad5-S vaccine, and the two-dose groups were primed with Ad5-S and boosted with Ad4-SE or Ad5-S. Vaccinated mice were challenged on day 42 with 1×10^4^ TCID_50_ SARS-CoV-2 via the intranasal route, after which lung and turbinate tissues were collected for viral gRNA detection on day 45 (**Figure 5A**). We observed that the IgG antibody and pNAb responses were significantly increased in all regimens after booster immunization, and the i.m.Prime-i.n.Boost regimen showed the highest fold induction (**Supplementary Figure S7**). Importantly, although serum antibody responses were significantly boosted after the second dose of immunization in the i.m.Prime-i.m. Boost regimen, protective efficacy on the respiratory tract in this group was not significantly better than that of the i.m. prime-only group, and even poorer than that of the i.n. prime-only group (**Figures 5B, C**). In contrast, the i.m.Prime-i.n.Boost and i.n.Prime-i.n.Boost regimens were more effective in protecting the lungs and turbinates, and Ad4-SE heterologous boost immunization completely protected the lungs, which was superior to the protective effects of Ad5-S (**Figures 5B, C**). These results once again emphasize the advantages of the Ad-based respiratory mucosal vaccines in defending against respiratory infections and prove the high protective efficacy of the Ad4-vectored vaccine in intranasal boost immunization.

## 4 Discussion

Adenoviral vectors are widely used for vaccine development, and Ad5-based vaccines for inhalation have been proven to be highly effective in preventing respiratory infections (29, 30). This implies the great potential of Ad-vectored mucosal vaccines in the prevention and control of respiratory pathogens. Considering the high PEI and the need to have a more effective immunization strategy when multiple administrations of the Ad-vectored vaccine are needed, we have developed a novel replication-deficient adenovirus type 4 vector whose protective efficacy and immunogenicity via a single-dose or heterologous immunization through the respiratory tract are superior to those of Ad5, making it an ideal adenovirus vector.

PEI can significantly reduce the immunogenicity of Ad-based vaccines (5, 31). This phenomenon may be attributed to the presence of pre-existing neutralizing antibodies against the viral vector in the serum (32), which may bind to the virus and prevent it from entering the cell to inhibit its ability to deliver antigens. Consequently, the development of adenoviral vectors with low PEI to alternative vaccine vectors is necessary. In our previous work, the percentage of people with moderate or high levels of anti-Ad4-neutralizing antibodies was less than 10%, which is much lower than the approximately 45% reported for Ad5 (25), and no cross-reactivity exists between anti-Ad5 NAbs and anti-Ad4 NAbs, indicating the advantage of Ad4 with significantly lower PEI. Furthermore, it has been reported that immune responses induced by the Ad4-vectored vaccine are not affected by Ad5 PEI in a high-dose model (26).

A primary challenge in the development of a novel Ad-vector is that the replication efficiency is generally low in HEK293 cells (27). In our study, the titer of the Ad4 vector with only an E1/E3 deletion was significantly lower than that of Ad5 (**Figure 1D**). Moreover, even the viral titer of Ad4 RI67 was lower than that of the replication-deficient Ad5 vector in HEK293 cells (**Figures 1F, G**). Much attention has been given to E4 modification to solve this problem (33, 34) because the E1B 55K protein of Ad5 interacts with the Ad5 E4 ORF6 protein to support adenovirus replication but shows suboptimal interactions with E4 from other types of Ad (35, 36). In our study, we proposed seven strategies for E4 modification and reported that the different modifications significantly influenced not only the viral titer but also the immune response (**Figures 1I–L**). Among them, we screened an Ad4 vector in which the E4 ORF4,6/7 gene was replaced with that of Ad5, which has the same growth kinetics and can induce an immune response comparable to that of the Ad5 vector.

Compared with Ad5, the majority of novel adenovirus vectors induce suboptimal immune responses (16–23). In our study, the antibody and cellular immune responses induced by the Ad4-vectored vaccine through the intramuscular route were comparable with those induced by the Ad5-vectored vaccine; however, when the Ad4-vectored vaccine was administered via the intranasal route, it induced a similarly robust response of serum pNAbs and mucosal antibodies and a significantly greater cellular immune response in the lungs than those of the Ad5-vectored vaccine (**Figure 2**). Consistent with these findings, Ad4-SE intranasal vaccination provided better protection in both the upper and lower respiratory tracts than Ad5-S. A single dose of Ad4-SE through the intranasal route provided complete protection to the lungs and turbinates, surpassing the protection provided by two-dose intramuscular immunization (**Figures 3B, C**, **5B, C**). These results suggest that the novel Ad4 vector has a high potential for vaccine development, especially for respiratory mucosal immunization.

The waning of vaccine efficacy over time and the emergence of new infectious diseases suggest that multiple-dose immunization with Ad-vectored vaccines may be needed (37, 38). In the prime-boost regimen, different types of adenoviruses and inoculation routes strongly influenced immune responses. The i.m.- or the i.n.-Prime combined with the i.n.-Booster regimen stimulated higher levels of pNAbs in the serum and superior protective mucosal antibody responses in the BALF, NLF, and lungs than the two-dose i.m. regimen (**Figures 4B–G**). Furthermore, in the Ad4 heterologous booster regimens, the serum antibody levels, T-cell responses in the spleen and lungs, mucosal antibody responses (**Figure 4**), and protective efficacy were all significantly greater than those induced by Ad5-based vaccine homologous immunization (**Figures 5B, C**). Among them, the Ad5 i.m.Prime-Ad4 i.n.Boost heterologous vaccination regimen induced the most optimal immune responses and showed the best protective efficacy.

A Zika vaccine based on the replication-deficient Ad4 vector with only E1-deletion was constructed in a previous study, which induced significantly lower cellular and humoral immune responses than Ad5 after intramuscular immunization (39). However, the Ad4 vector with a different design in our study showed better immunogenicity than Ad5. Comparing the two studies, the antigen expression level of the Ad4-vectored Zika vaccine was lower than that of the Ad5 vector in cells (39), but the antigen expression levels of the Ad4 vector in our study were comparable in HEK293 cells and higher in A549 cells than those of the Ad5 vector. Different modification strategies result in different antigen expression capacities, and there is a significant positive correlation between the immunization efficacy and antigen expression levels (21), which may account for the different immunogenicity between the Ad4 Zika vaccine and ours.

Safety concerns related to Ad-vectored vaccines attracted much attention during the SARS-CoV-2 pandemic. Vaccine-induced thrombotic thrombocytopenia (VITT) was found in a minority of AZD1222 recipients (40), a lower incidence was observed in recipients of Janssen HAdV-D26.COV2. S vaccine (41), and few cases associated with Ad5 vaccination were reported (42). The occurrence of VITT differs among Ad serotypes; adenoviruses from non-human subgroups may carry greater risks when used, and human Ad serotypes with natural tropism to respiratory tracts may be safer and more suitable for the development of respiratory mucosal vaccines. Ad4 is one of the leading adenovirus types that cause acute respiratory infections. Notably, millions of military recruits have been vaccinated with the live wild-type Ad4 (43), with a favorable safety profile in the U.S. military since 1971. A clinical trial of replication-competent Ad4-based H5N1 resulted in no severe adverse responses (44). These backgrounds suggest that replication-deficient Ad4 may have the lowest safety risk in the development of rare-serotype adenovirus vectors. Our study demonstrates that Ad4- and Ad5-vectored vaccines exhibit comparable biodistribution profiles post-immunization; however, when immunized intranasally, the Ad4 vectored recombinant vaccine demonstrates a superior immune response. The immune mechanisms of the different immunogenicity need to be further investigated.

In conclusion, the new replication-deficient Ad4-vector had a high yield and antigen expression. Compared with Ad5, the Ad4 vector induced a superior immune response and better protection via an intranasal single-dose regimen. In addition, the humoral, cellular, and mucosal immunity of the Ad4-vector heterologous booster immunization was significantly superior to that of the homologous booster of the Ad5 vector. The novel Ad4 vector constructed in this study offers a new option for vaccine development.

## Data Availability

The datasets presented in this study can be found in online repositories. The names of the repository/repositories and accession number(s) can be found in the article/**Supplementary Material**.
