# Supplementary material for: Development of a novel adenovirus type 4 vector as a promising respiratory vaccine vehicle
Source: Front Immunol. 2025 Apr 10;16:1572081. doi: 10.3389/fimmu.2025.1572081 (PMC12018414; doi:10.3389/fimmu.2025.1572081)
Supplement: Supplementary file 1 [file DataSheet1.docx]

Supplementary Materials for

Development of a novel adenovirus type 4 vector as a promising vaccine respiratory vehicle

Jinghan Xu^1,#^, Zhenghao Zhao^1,#^, Shipo Wu^1^, Zhe Zhang^1^, Shuling Liu^1^, Nan Huo^1^, Wanru Zheng^1^, Yi Chen^1^, Zhiqiang Gao^1^, Zuyuan Jia^1^, Tianyu Liu^1^, Busen Wang^1,^*, Li Zhu^1,^*, Lihua Hou^1,^*

*Correspondence: [houlihua@sina.com](mailto:houlihua@sina.com) (L. Hou), [jewly54@bmi.ac.cn](mailto:jewly54@bmi.ac.cn) (L. Zhu), [sen154034@163.com](mailto:sen154034@163.com) (B. Wang)

**This file includes:**

Figures. S1 to S7


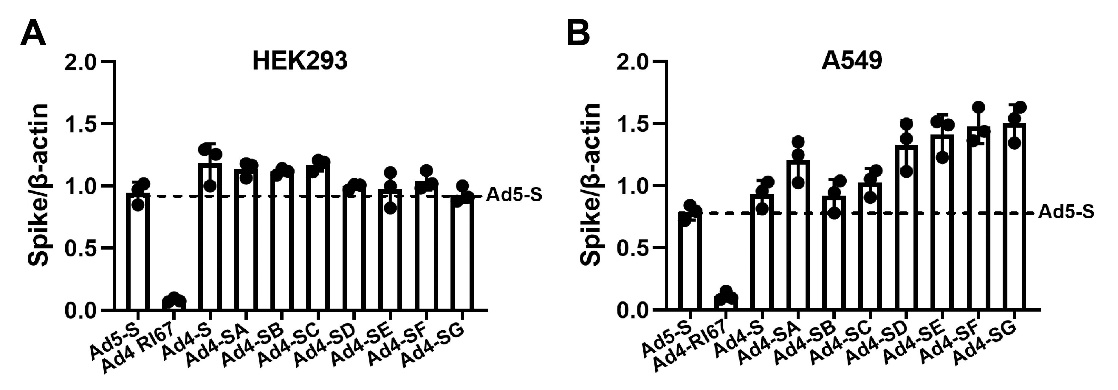


**Supplementary Figure S1. The gray levels of the indicated protein.** HEK293 (A) and A549 (B) cells were infected with Ad5-S, Ad4 RI67, Ad4-S, and Ad4-SA to SG at MOI of 1. The transgene and β-actin expression levels were validated through western blotting and quantified the gray levels of the indicated protein. Respective ratio of the gray value of transgene to β-actin expression levels. Data was shown mean ± SEM values.

**
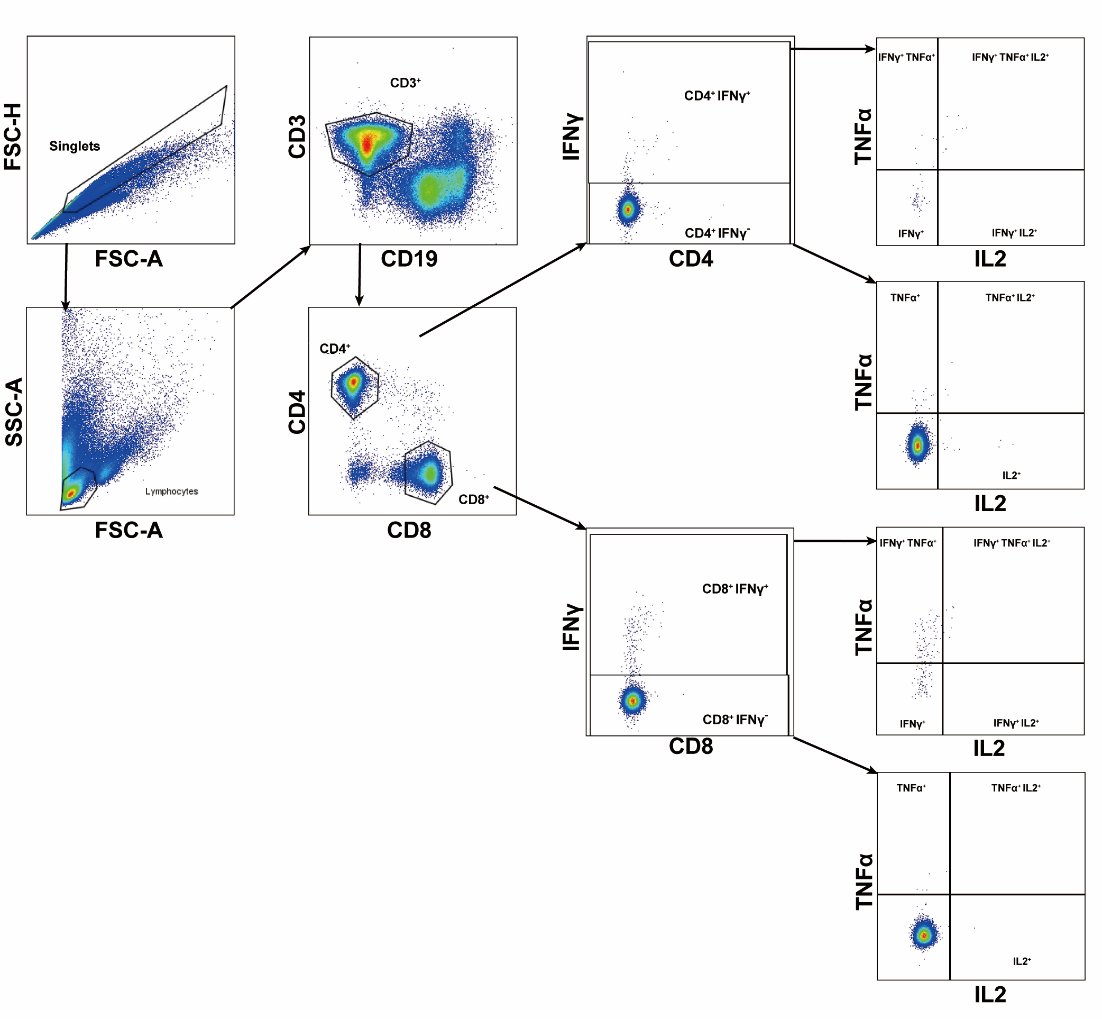
**

**Supplementary Figure S2. Flow Cytometric Gating strategies.** Flow gating strategies for the identification and quantification of SARS-CoV-2-specific CD4^+^ and CD8^+^ T cells.
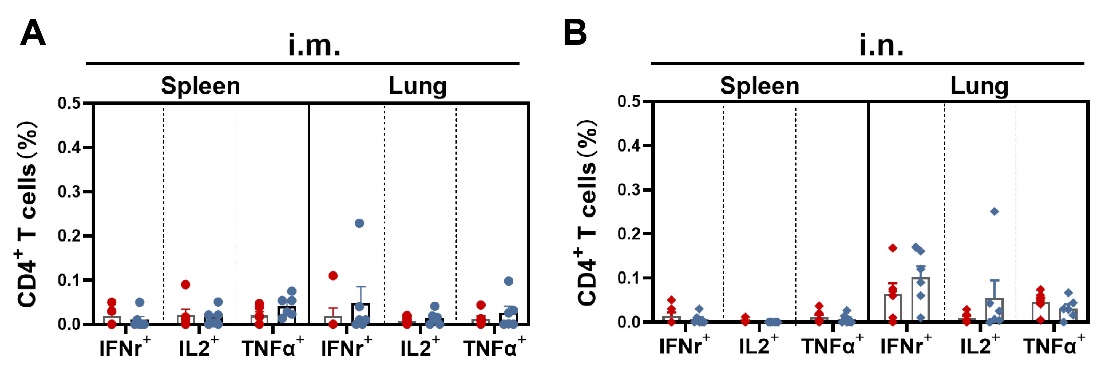


**Supplementary Figure S3** **CD4^+^ T-cell response of single-dose immunization with Ad4-SE or Ad5-S in the spleen and lungs.** IFNγ, IL2, TNFα expressing CD4^+^ T-cell responses in the spleen and lungs were measured by flow cytometry in the i.m. (A) and i.n. (B) groups. Data was shown mean ± SEM values. **
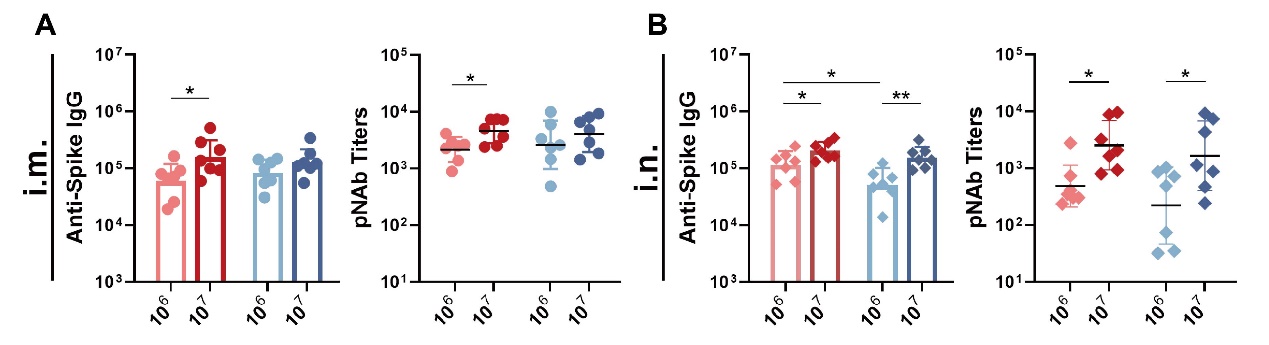
**

**Supplementary Figure S4 The antibody responses induced by single-dose vaccination with Ad4-SE or Ad5-S in hACE2 transgene mice**. The anti-spike IgG titers and pNAbs titers in i.m. (A) and i.n. (B) groups before SARS-CoV-2 challenge. The bar graphs show the GMT values with 95% CI, statistical significance among the different groups was determined by two-tailed t-test.


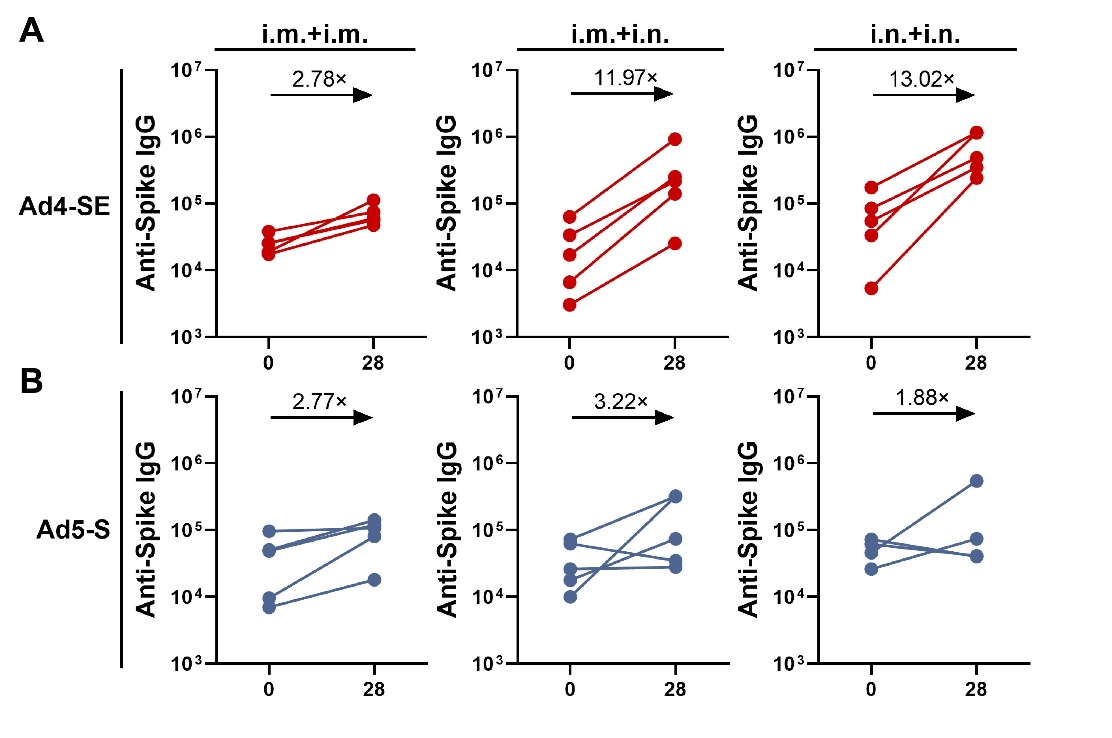


**Supplementary Figure S5. Heterologous immunization of Ad4-SE induced superior antibody response with the extended time interval between priming and boosting.** BALB/c mice (n = 5 per group, one mouse was found dead in the i.n.Prime-i.n.Boost regimen with Ad5-S) were boosted with Ad4-SE (A) or Ad5-S (B) (with 1×10^6^ IFUs dose) at interval of 18 months after Ad5-S priming. The anti-Spike IgG binding antibodies were determined on day 28 post-boosting.


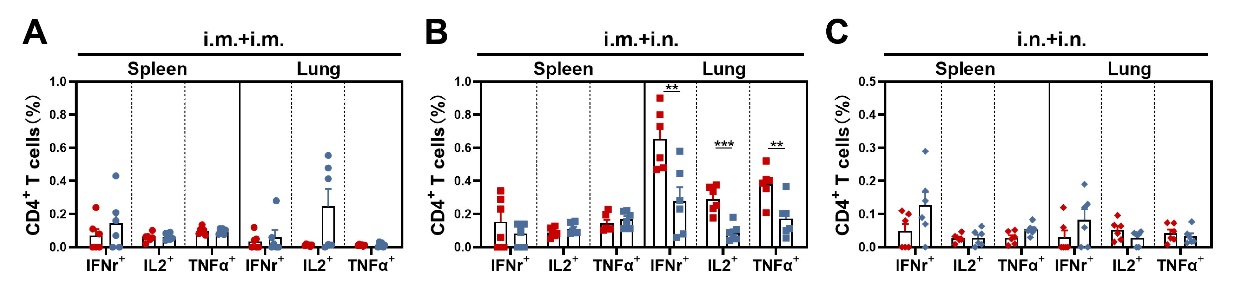


**Supplementary Figure S6 CD4+ T-cell response of Ad5-S-prime and boost with Ad4-SE or Ad5-S immunization in spleens and lungs.** IFNγ, IL2, TNFα expressing CD4+ T-cell responses (A) in spleens and lungs were detected in i.m.Prime-i.m.Boost route and in i.m.Prime-i.n.Boost route (B) and in i.n.Prime-i.n.Boost route (C). Data was shown as mean ± SEM, statistical significance was determined by two-tailed t tests.


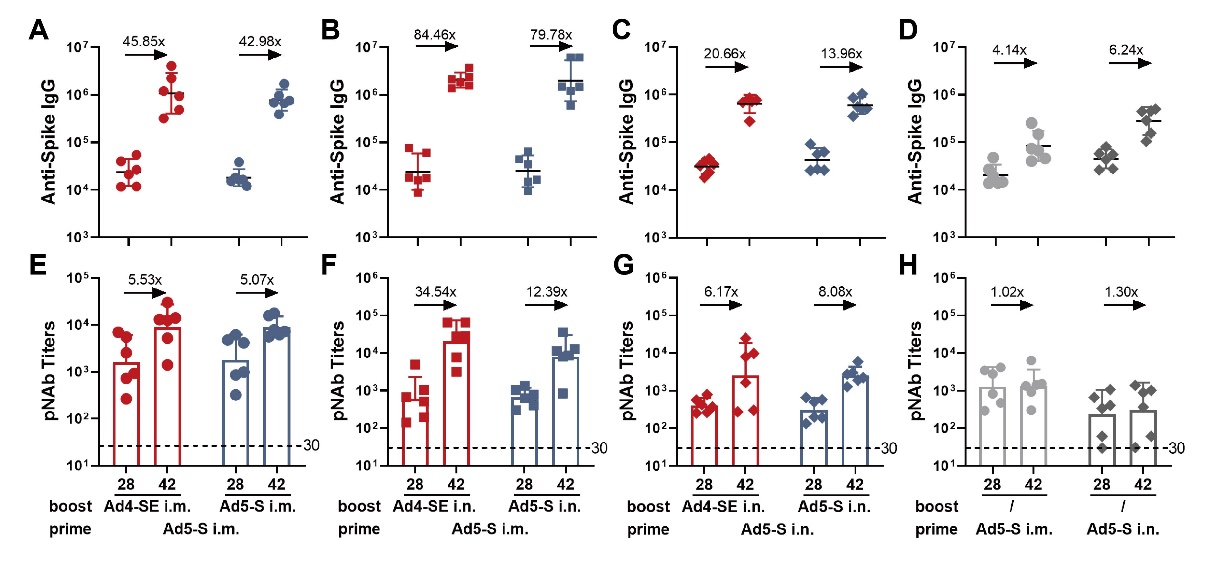


**Supplementary Figure S7 Ad4-SE heterologous booster vaccination provided superior antibody responses in hACE2 transgene mice**. The anti-Spike IgG titers and pNAbs titers on day 28 and day 42 in i.m.Prime-i.m.Boost groups (A, E), i.m.Prime-i.n.Boost groups (B, F), i.n.Prime-i.n.Boost groups (C, G), and the i.m. or i.n. prime-only groups (D, H). The bar graphs show the GMT values with 95% CI, statistical significance was determined by one-way ANOVA with Tukey’s multiple comparisons tests among the different prime-boost regimens and by two-tailed t test between Ad4-SE and Ad5-S.
